# Evidence-based practice: beliefs, attitudes, knowledge, and skills among Colombian physical therapists

**Published:** 2015-03-30

**Authors:** Robinson Ramírez-Vélez, Jorge Enrique Correa-Bautista, Diana Isabel Muñoz-Rodríguez, Lorena Ramírez, Katherine González-Ruíz, María Andrea Domínguez-Sánchez, Diana Durán-Palomino, Montserrat Girabent-Farrés, María Eugenia Flórez-López, M Caridad Bagur-Calafat

**Affiliations:** 1 Grupo GICAEDS, Facultad de Cultura Física, Deporte y Recreación, Universidad Santo Tomas, Bogotá, Colombia; 2 Centro de Estudios en Medición de la Actividad Física (CEMA), Escuela de Medicina y Ciencias de la Salud, Universidad del Rosario, Bogotá, Colombia; 3 Programa de Fisioterapia, Facultad de Salud, Universidad CES, Medellín, Colombia; 4 Instituto Universitario de Ciencias de la Salud, Fundación H.A. Barceló, Buenos Aires, Argentina; 5 Grupo de Ejercicio Físico y Deportes, Programa de Fisioterapia, Facultad de Salud, Universidad Manuela Beltrán, Bogotá, Colombia; 6 Universidad de La Sabana, Campus Universitario del Puente del Común, Chía, Colombia; 7 Departamento de Fisioterapia, Universitat Internacional de Catalunya, Barcelona, España

**Keywords:** Physical therapy, evidence-based practice, research, attitude, clinical practice

## Abstract

**Objective:**

The main purpose of this study was to describe a group of Colombian physical therapists' beliefs and attitudes towards Evidence-Based Practice (EBP), their education, knowledge and skills for implementing EBP, the use of relevant literature in clinical practice, access to and availability of scientific information and perceived barriers to including EBP in practice.

**Methods:**

This was a cross-sectional study which involved 1,064 Colombian physical therapists. The study used a 50-item screening questionnaire EBP developed to estimate attitudes, beliefs, knowledge and skills regarding. This instrument has been adapted and was validated previously in Colombia by Flórez-López *et al*.

**Results:**

The population mostly consisted of young females (77.2%) aged 22 to 29 years old (79.4%). Most respondents had an undergraduate degree (87.7%). The physical therapists stated that they had positive attitudes and beliefs regarding EBP, most of them answering that they agreed or strongly agreed that EBP is necessary (71.6%), the relevant literature is useful for practice (61.3%), EBP improves the quality of patient care (64.1%) and evidence helps in decision-making (44.5%). Forty-one percent of the respondents indicated that a lack of research skills was the most important barrier to the use of evidence in practice.

**Conclusion:**

The physical therapists reported that they had a positive attitude to EBP and were interested in learning about or improving the skills necessary to adopt EBP in their clinical practice.

## Abstract (es)

**Objetivo:**

Describir en un grupo colombiano de fisioterapeutas las i) creencias y actitudes hacia la practica basada en la evidencia (PBE), ii) la educación, el conocimiento y las habilidades para implementar la PBE; iii) el uso de la literatura relevante en la práctica clínica; iv) el acceso y la disponibilidad de información científica; y v) la percepción de las barreras para la inclusión de la PBE.

**Métodos:**

Se realizó un estudio transversal en 1,064 fisioterapeutas colombianos. El estudio usó el cuestionario que consta de 50-ítems para estimar las actitudes, creencias, conocimientos y habilidades hacia la PBE. Dicho instrumento fue adaptado y validado en Colombia por Flórez-López *et al*.

**Resultados:**

La mayoría de la población participante fueron mujeres (77.2%) en edades comprendidas entre 22 y 29 años (79.4%). El 87.7% de los encuestados eran titulados en fisioterapia. Los fisioterapeutas manifestaron tener actitudes y creencias positivas hacia la PBE. Una mayoría respondió que estaba de acuerdo o muy de acuerdo en que es necesaria la PBE (71.6%), en que la literatura es útil para la práctica clínica (61.3%), que la PBE mejora la calidad de la atención a los pacientes (64.1%), y en que la evidencia ayuda en la toma de decisiones clínicas (44.5%). El 41.0% de los encuestados indicaron que la falta de habilidades de investigación era la barrera más importante para el uso de la evidencia científica en la práctica clínica.

**Conclusiones:**

Los fisioterapeutas manifestaron una actitud positiva acerca la PBE y estaban interesados en aprender o mejorar las habilidades necesarias para adoptar la PBE en la práctica clínica.

## Introduction

Incorporating research results into clinical practice assumes that a strategically approach must be adopted by anyone working in healthcare seeking to achieve acceptable levels of effectiveness 1,2. Regarding the above, different authors have suggested incorporating evidence-based practice (EBP), defined as being a process whose objective is selecting the best scientific arguments for resolving problems encountered in daily clinical practice. EBP implies integrating individual clinical experience with the best available external scientific evidence regarding systematic investigation and, on occasions, its inclusion requires change regarding practice, self-directed learning and a favourable work environment 3,4.

Few studies has been carried out to date by Latin-American physiotherapists concerning EBP-related knowledge, practice and attitudes as most studies have been focused on examining the use of evidence, including critical reviews of studies and using scientific literature in clinical practice 5. Nevertheless, the teaching, evaluation and use of EBP in daily clinical practice have been included in study plans in countries like Australia 6, Spain 7, the USA 8 and the UK 9.

This study's main objective was thus to describe a group of Colombian physiotherapists' beliefs and attitudes regarding EBP, the necessary education, learning and skills for implementing EBP, using the relevant literature regarding clinical practice, access to and the availability of scientific information and perception of barriers against incorporating EBP.

## Materials and Methods

### Participants

Graduates in physiotherapy (lacking specialised training in EBP) were selected from nine cities in Colombia. The inclusion criteria consisted of having a BSc degree in physiotherapy and consenting to participate in the study. A structured survey was designed using on-line Survey Monkey (https://www.surveymonkey.com/s/JHNV89J) for obtaining the sample; this contained EBP questionnaire validated on a Colombian population by Flórez-López *et al* 10. The data was collected from February 2012 to June 2013; 1,250 visits were made during this time. There were 1,064 valid data entries (85% of the total visits). 

### Instrument

The authors of this study used a questionnaire developed in the USA by Jette *et al*. 8, and validated in Spanish by Guerra *et al*.^7^, for determining nursing and allied health professions' attitudes, beliefs, skills, learning, behaviour and barriers concerning EBP. This questionnaire was structured in sub-dimensions via a series of items grouping and collecting information related to each of these categories. Items 1, 2, 4 and 6-11 collected data about attitudes and beliefs concerning EBP whilst items 3 and 5 measured interest and motivation regarding putting EBP into effect. Academic background, learning and skills related to access to and interpretation of scientific information were covered by items 18, 19 and 21-23. Item 32 dealt with perceived barriers regarding using EBP whilst items 15-20 were related to qualified physiotherapists' use of and access to clinical practice guidelines. Items 33-50 collected demographic information and data about the qualified physiotherapists who answered the questionnaire. The structure of the items collecting data about EBP-related attitudes, beliefs, education, learning and skills was formulated using a 0 to 4 ordinal scale, following the adaptation for the Spanish version where "strongly agree" and "strongly disagree" had maximum and minimum scores, respectively. Other items collecting data about access to information used a dichotomous response: "yes" or "no". The questions related to the understanding scientific concepts related to EBP required a multiple option answer having three possible choices: "I fully understand", "I partially understand" or "I do not understand".

### Procedure

The EBP-related study involving Colombian physiotherapists was implemented via a web page; it was disseminated through different actions via Internet through various Colombian Higher Education institutions' further education programmes. A promotional digital banner was created for disseminating the survey through social networks. A presentation describing the study's objective was given before a person would anonymously begin the questionnaire. It urged such person to answer each question honestly and informed consent was requested before continuing. A user then began completing the questionnaire which was programmed to avoid missing values. This study was approved by Universidad Manuela Beltrán's Ethics Committee for research involving human beings (Resolution N° 1008-2012-014), following the deontological standards recognised by the Declaration of Helsinki and current Colombian law regulating research involving human beings (Ministry of Health Resolution 008430/1993).

### Statistical analysis

The information gleaned from the questionnaires was typed on an Excel ® spreadsheet (Windows 8®) and the Statistical Package for the Social Sciences ® (version 21.0, Chicago, IL, USA) was used for processing it. Normally distributed data was reported as absolute and relative frequency. Bivariate analysis using the *X* ^2^ test was used for evaluating the association between the participants' overall characteristics and the categories in Jette *et al*.´s EBP questionnaire 8.

## Results

### The participants characteristics 

Sample consisted of 1,064 physiotherapists, 821 of whom were female (77.2%) and 243 male (22.8%). Most people surveyed were aged 20-29 years-old (845 participants: 79.4%). The other characteristics are shown in Table 1.

**Table 1.**
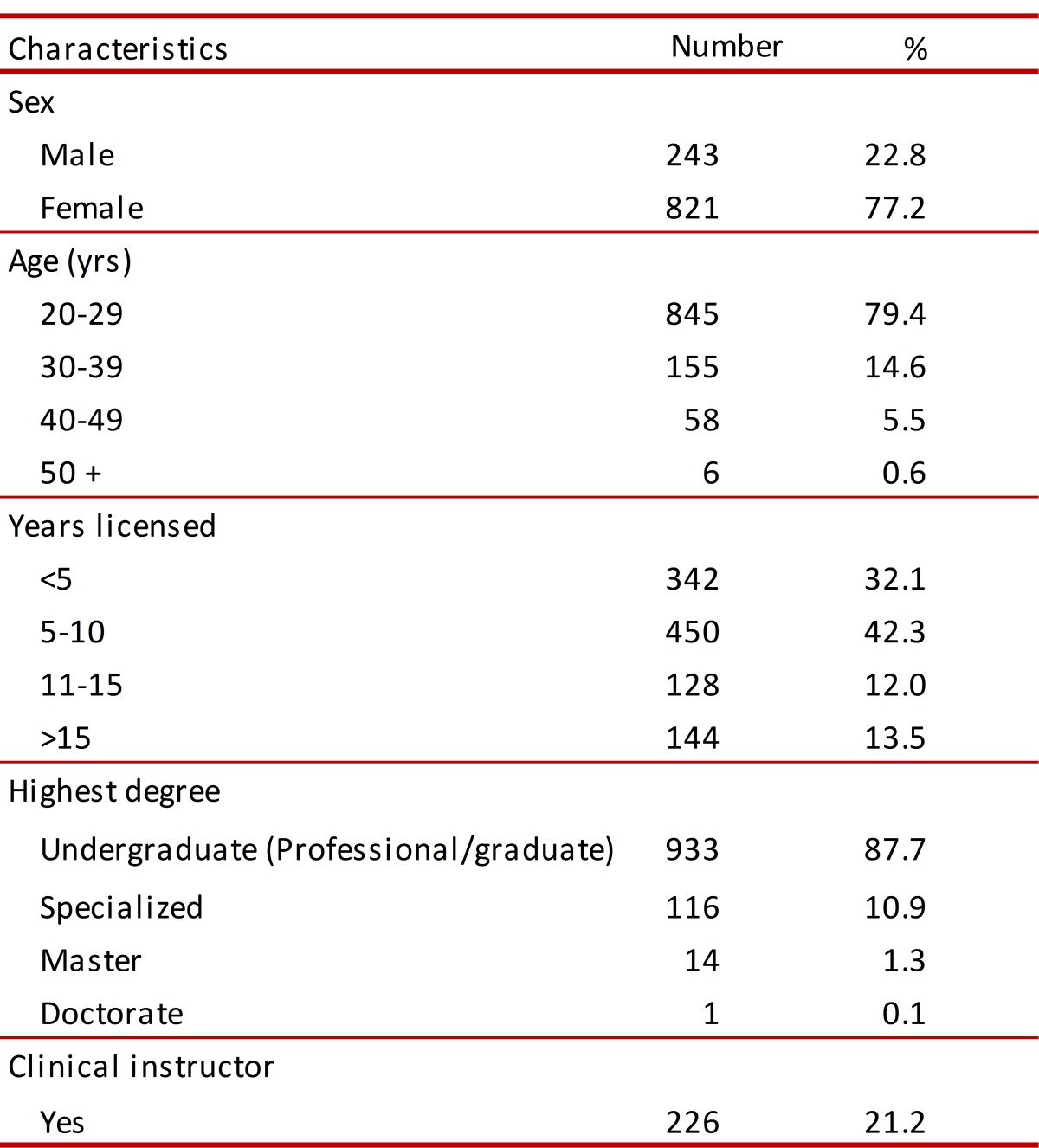
Characteristics of Respondents.

### Characteristics regarding clinical practice

Regarding the characteristics of engaging in clinical practice for the physiotherapists participating in the study, it was established that most of them worked 31-40 hours weekly. The largest amount of patients seen per day was 5-10 patients (30.7%) and most of those surveyed stated that their clinical activity was accompanied by (at least) 5 qualified physiotherapists (39.2%). Cardiovascular and respiratory alterations represented the most frequently occurring reason for consultation by patients receiving attention. The other characteristics regarding healthcare practice are given in Table 2.

**Table 2.**
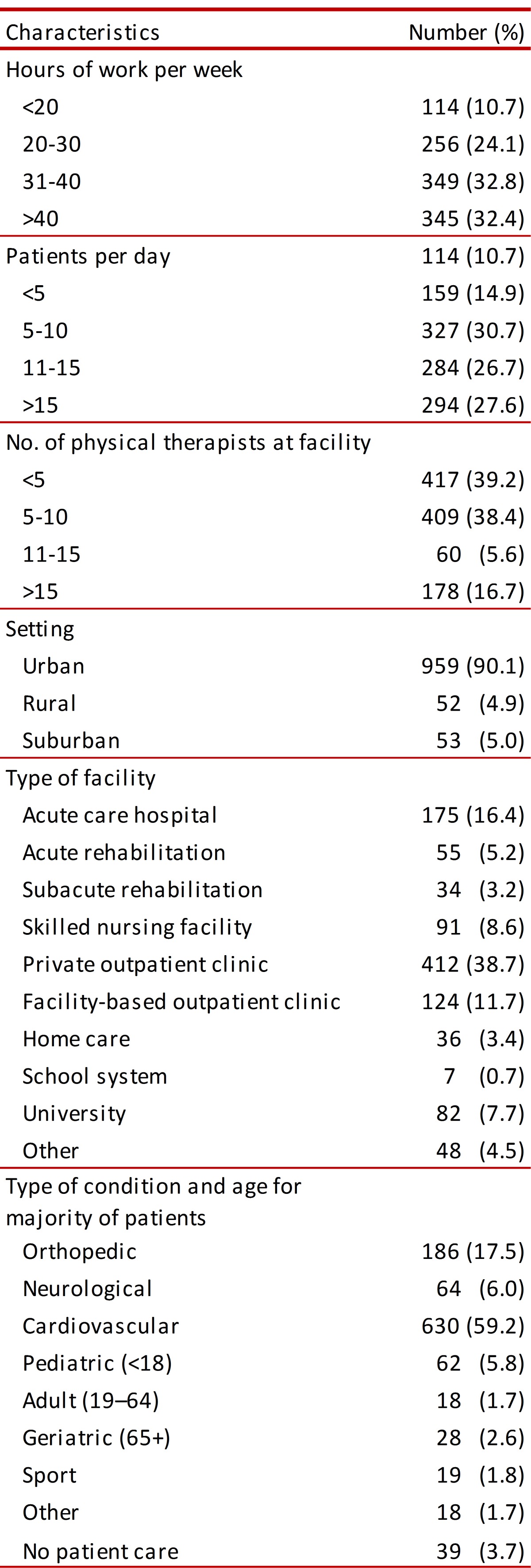
Characteristics of Respondents' Practice.

### Attitudes and beliefs

Seventy-one percent of those surveyed strongly agreed and 27.4% agreed that using EBP was necessary when practicing physiotherapy. Sixty-one percent stated that they strongly agreed that the literature and findings regarding research were useful in daily clinical practice, whilst 64.1% of those surveyed stated that they strongly agreed that EBP improved quality regarding attending patients. Decision-making concerning patient care was catalogued as a useful tool by 94.5% of those surveyed. Evidence-based practice did not imply a higher income for 45.4% of the physiotherapists. The other results are given in Table 3.

**Table 3.**
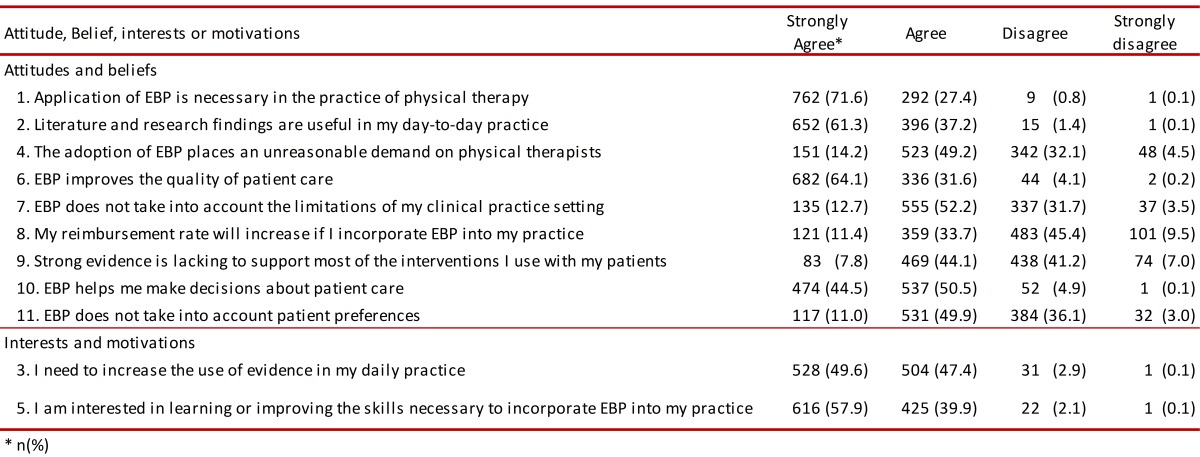
Self-reported attitudes, beliefs, interests and motivations about EBP.

###  Interests and motivation

Regarding the need for increasing the use of scientific evidence concerning EBP in daily clinical practice, 49.6% of those surveyed stated that they strongly agreed and 47.4% agreed. Likewise, 57.9% stated that they strongly agreed with learning or improving skills for incorporating EBP into daily clinical practice. The other results are given in Table 3.

###  Using tools for applying EBP

Reading and reviewing scientific literature as a tool for incorporating EBP meant a monthly reading of 2-5 articles by 53.1% of those surveyed. Clinical decision-making supported by scientific literature and associated findings was practised by 49.6%. Regarding the use of PubMed/MedLine and other databases when searching for relevant articles or findings concerning practice, 39.7% did so 2-5 times per month, whilst 5.5% did so more than 16 times per month (Table 4).

**Table 4.**
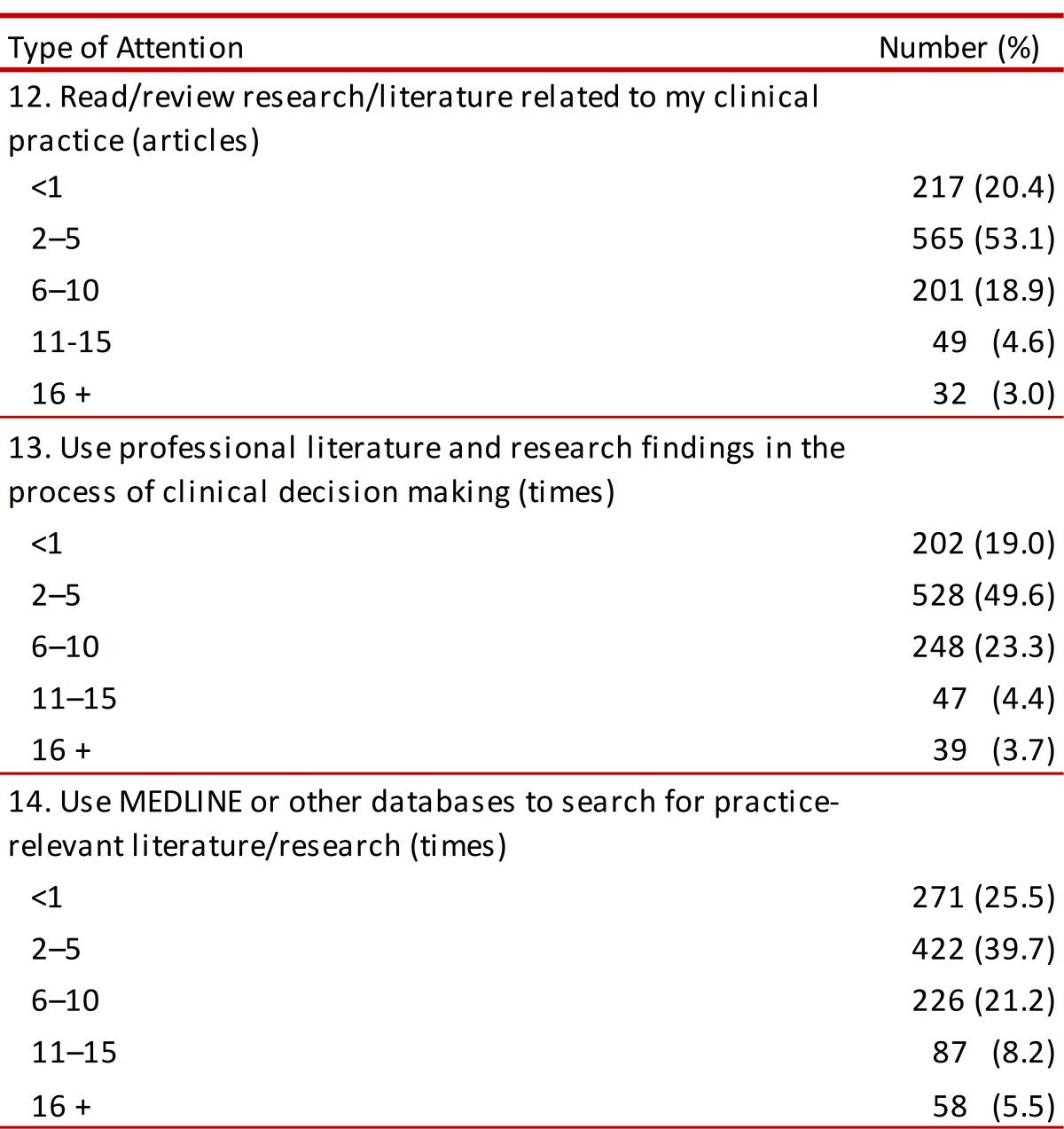
Self-reported use about EBP.

###  Applying EBP tools and access to them

It was found that learning about the availability of EBP tools was managed by 69.9% and unknown by 14.5%. Learning about the existence and availability of on-line guidelines was reported by 78.1% of those surveyed, whilst 21.9% stated that they did not know about this information. Using relevant databases and Internet at home or in places different to the workplace was an available tool for 86.9% of those surveyed (Table 5). 

**Table 5.**
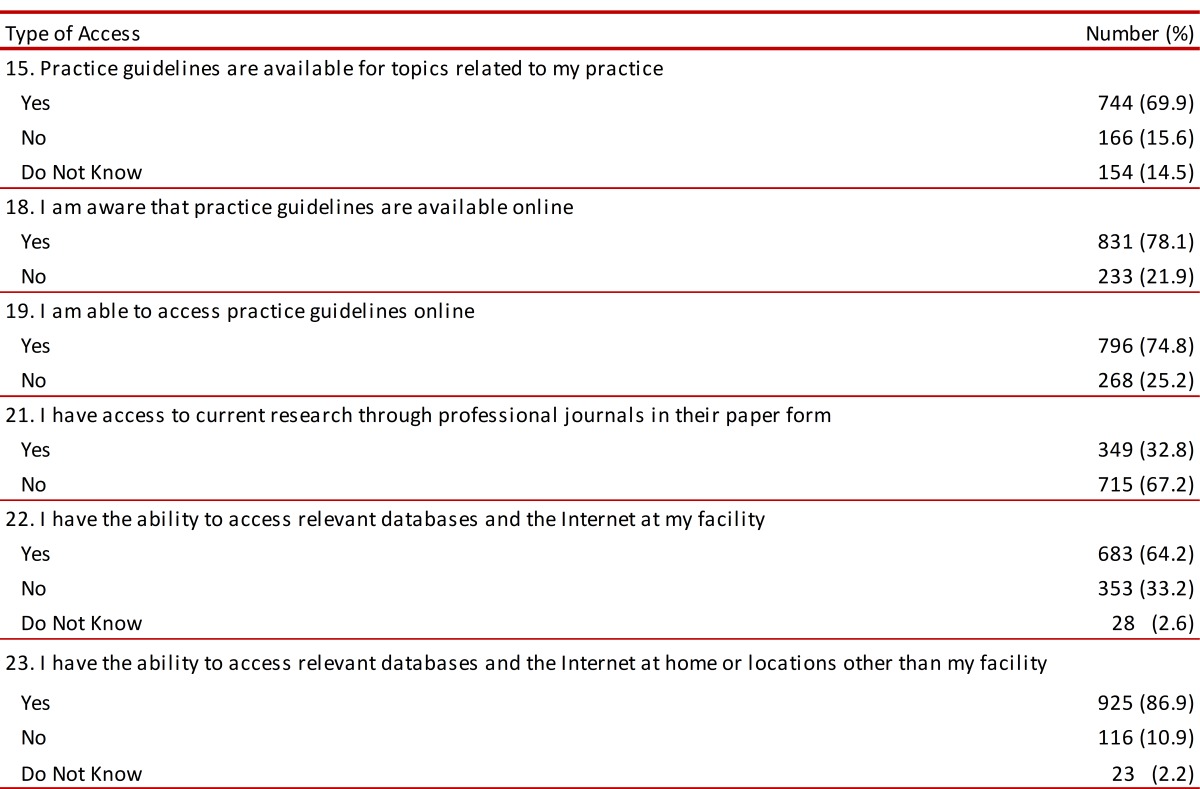
Application tools and access to EBP in Colombian Physiotherapists.

###  Using EBP tools and learning about them

Sixty-three percent of those interviewed stated that they used EBP tools. Regarding institutional support concerning the workplace for incorporating research findings as part of clinical practice, 47.2% stated that they agreed whilst 32.2% stated that they strongly agreed. Eighty-eight percent of those surveyed stated that they had received basic teaching regarding EBP during their academic degrees, although only 48.9% of those participating in the study stated that they were familiar with specific bibliographic search engines (Table 6).

**Table 6.**
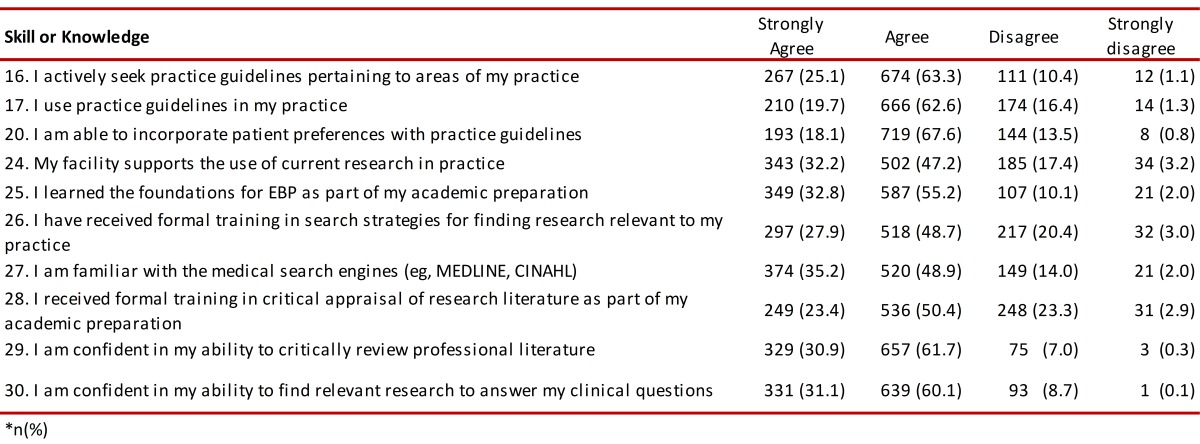
Knowledge and use of tools for EBP.

###  Self-reported access to and availability of literature

**Figure 1.**
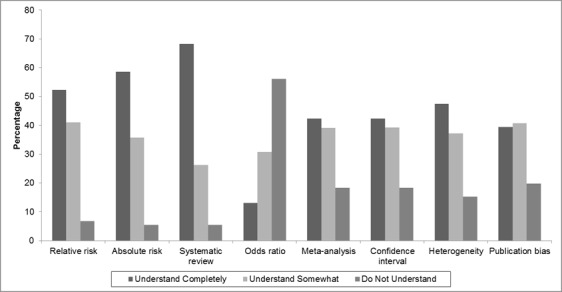
Self-reported access to and availability of literature.

More than 50% of respondents stated that they fully understood the terms *systematic review *(68.2%), *absolute risk *(58.6%) and *relative risk *(52.3%). On the other hand, the terms least understood by those surveyed were *publication bias* (19.8%), *meta-analysis* and *confidence interval *(18.4%)*, heterogeneity (*15.2%) and *odds ratio *(13.1%) (Fig. 1). 

###  Self-reported ranking of barriers to evidence-based practice

**Figure 2.**
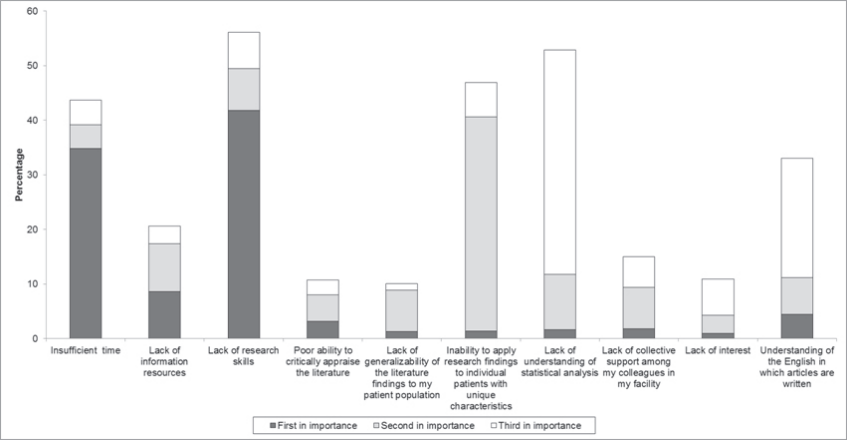
Self-reported ranking of barriers to evidence-based practice.

Forty-one percent of those surveyed stated that a lack of research skills was the most important barrier to using EBP. Thirty-nine and forty-one percent of those surveyed stated that the second and third barriers, respectively, were a lack of understanding of statistical analysis and an inability to apply research findings to individual patients with unique characteristics. Understanding of the English in which articles are written was stated as being a significant barrier for 21.9% of those surveyed (Fig. 2).

###  Relationship between the participants' characteristics and EBP questionnaire categories 

A bivariate study of the participants' characteristics and the categories of Jette *et al*.´s EBP questionnaire 9 revealed that variables such as age, how many years since graduation, the average of hours service per week and the number of patients attended per day gave a statistically significant (*X* ^2 ^ <0.001) OR >1. It was also seen that qualified physiotherapists who had obtained their BSc in physiotherapy 11-15 years ago and more than 30 years ago stated that most "agreed" with the advantages of incorporating EBP in their clinical practice. Physiotherapists having a BSc in Physiotherapy working fewer hours per week and/or attending fewer patients per day stated that they "strongly agreed" that EBP helped in decision-making regarding patient care.

##  Discussion

Several studies 1-9, 11,12, have examined using EBP in a particular profession and its relationship with the type of education received during undergraduate or postgraduate studies. The barriers limiting EBP use in a profession must be identified so as to propose strategies facilitating EBP use once a person has qualified as a physiotherapist. Manspeaker *et al*. 13, have shown that students trained in using the EBP model from undergraduate studies onwards have increased their learning, attitude, security and clarity regarding scientific concepts. However, implementing EBP could be limited by some aspects involved in care practice, such as that reported recently by Dannapfel *et al* 14. Their research involving a group of Swedish physiotherapists showed that personal aspects such as employment conditions, search methods, reading scientific evidence and a lack of time could limit EBP use.

###  Interests and motivation

The present study's results showed that most physiotherapists surveyed stated their interest in improving their skills for incorporating EBP in their practice as qualified physiotherapists; such finding coincided with that reported by Jette *et al*. 8, and Guerra *et al*.^7^, as both authors identified that EBP use by healthcare personnel was necessary and useful in decision-making regarding healthcare practice. Regarding Latin-America, Durán-Palomino *et al*. 3, examined compliance with British Thoracic Society (BTS) recommendations for cystic fibrosis in 7 regions in Colombia. The results showed that, although qualified physiotherapists mentioned knowing about the importance of EBP as a strategy for maintaining high standards of clinical care, the qualified physiotherapists surveyed preferred to use the traditional model of clinical intervention 15,16.

###  Beliefs and attitudes regarding EBP

Our results showed that qualified Colombian physiotherapists considered that EBP is necessary and useful in clinical practice and leads to improving care quality; such findings coincide with that found regarding physiotherapists by other authors 3,7-9 and concerning other professions 17,18. It was also found that a large percentage of those surveyed, who stated that scientific EBP was adopted in their work, needed time for searching for data, analysing it and using/incorporating it. Such "time" factor is a significant limiting factor regarding EBP use, since most physiotherapists surveyed had a high workload, attending an average of 11 or more patients per day and working an average 31 or more hours per week. Nevertheless, work by Aarons *et al*., in the USA 19, has shown the need for integrating some factors for using EBP, such as a qualified physiotherapist's attitude, postgraduate training and clinical context which, together with scientific associations and networks, should guarantee that qualified physiotherapists could use EBP effectively.

### Using tools for applying EBP

Regarding the use of clinical practice guidelines as a tool for aiding decision-making, it was found that most of those surveyed stated that they were able to incorporate guidelines and recommendations regarding EBP into their practice as qualified physiotherapists. Nevertheless, efforts must continue to be made regarding the implementation and use of such available tools for EBP to reduce uncertainty concerning taking clinical decisions affecting physiotherapy. Regarding these observations, the American Physical Therapy Association (APTA) has proposed strengthening EBP use through methodological strategies, such as the use of clinical cases or problem-based learning, seeking to motivate physiotherapists regarding this practice. Even though there is sufficient motivation for learning about and going deeper into EBP, aspects affecting its implementation must be considered, such as the healthcare services' care model, quality control and the lack of evidence in some pathologies supporting a physiotherapist's intervention.

Previous studies by Ramírez-Vélez *et al*. 1,2, and Durán-Palomino *et al*. 3, in Colombia has identified four essential limitations concerning using tools for implementing EBP: evidence-based guideline use in clinical institutions, little time for preparing and evaluating the available evidence when qualified physiotherapists were working in care centres, the late arrival of effective evidence in clinical practice and being unaware of technological applications for searching for and using the best available evidence in care practice 11.

###  Self-reported knowledge of specific terms 

There was an abundance of conceptual and methodological knowledge about research design concerned with self-directed learning (SDL) about statistical aspects and measurements of association and impact. However, some deficiencies were reported regarding concepts which would allow classifying the quality of evidence, such as heterogeneity, confidence intervals, meta-analysis and bias. It could be said that SDL, as shown by the qualified physiotherapists surveyed about statistical aspects, accounted for limited learning when evaluating the best available evidence 1-3,11.

### Self-reported ranking of barriers to evidence-based practice

As in other work, the main obstacle for implementing EBP was a lack of research skills 1,8. The results coincided with other work showing that a lack of standardised processes regarding preparing tools for the systematic search for the best available evidence in Colombia is subject to interventions involving highly variable clinical care 3,11. Adapting high-quality evidence-based clinical practice processes seems to be an effective and efficient option for many healthcare organisations, since this would avoid unnecessary duplication of efforts. Nevertheless, having EBP resources or tools available in healthcare centres does not guarantee that they will be used, given that implementing them will depend on rating potential organisational barriers when applying them, this having to do with applicability offering a list of key criteria for monitoring results and/or auditing them 20.

Regarding the study's limitations, on-line access to the platform should be noted. The instrument and the variables used in this study were selected following a review of the pertinent literature. However, just as with any other study, we included just a finite number of questions. Another limitation is concerned with participants' personal characteristics not being taken into account as work by several authors has shown that they significantly modulate an inclination to apply EBP 11. Decision-making requires knowing about and managing the elements and stages shaping EBP, as well as the barriers and strategies presented during implementing it. However, such limitations do not compromise the results obtained when validating them. 

Concerning study strengths, it is worth highlighting that this is one of the first studies in Colombia explicitly describing the EBP conceptual framework applied to graduates having a BSc in Physiotherapy. Even though the variability found in this study does not necessarily imply that Colombian physiotherapists are using EBP inappropriately in care practice, this can be determined by studies involving mixed designs and approaches. This type of evaluation can only be used if there is agreement on its use, ideally regarding how to apply clinical practice-based guidelines. Even though making clinical decisions involves a personal dimension by those taking them, most carry out their activity within healthcare services' regulatory and organisational framework 12,15,16,21. 

Colombian physiotherapists thus stated their positive attitude towards EBP and were interested in learning about or improving the skills needed for adopting EBP in clinical practice.​ Nevertheless, a lack of research skills, learning regarding methodology and statistics were revealed as some of the main obstacles for the implementing EBP. 
